# Assessment of School Contributions to Healthy Eating, Physical Activity Education, and Support for Weight-Loss Attempts among Adolescents from Jazan, Saudi Arabia

**DOI:** 10.3390/nu15214688

**Published:** 2023-11-05

**Authors:** Ibrahim M. Gosadi

**Affiliations:** Department of Family and Community Medicine, Faculty of Medicine, Jazan University, Jazan 82621, Saudi Arabia; gossady@hotmail.com

**Keywords:** adolescents, body weight, school, healthy eating, physical activity, weight loss, Jazan, Saudi Arabia

## Abstract

This study evaluates the contribution of schools in Saudi Arabia towards the provision of education and support concerning weight management among adolescents. It also identifies adolescents who have attempted to lose weight and examines their preferred weight-loss methods and their sources of weight-loss support. This study is a cross-sectional investigation that targets adolescents in intermediate and secondary schools in Jazan, Saudi Arabia. Data was collected via a self-administered questionnaire that looked at the involvement of the school in the provision of education pertaining to weight control and the weight-loss experiences of students who had attempted to lose weight. The study involved 501 adolescents, 45% of whom had attempted to lose weight, where the most common methods for weight loss being reducing food consumption (77%), exercising (75%), and fasting (49%). The assessment of the schools’ contributions indicated its suboptimal role in the provision of education concerning physical activity compared to education about eating habits. The most commonly reported sources of weight-loss support were the students’ families (54%), followed by community facilities (44%). The least common sources were the students’ schools (24%). These findings indicate that schools need to enhance their contribution to encouraging adolescents to adopt a healthy lifestyle, while also ensuring multi-sectorial collaboration with families and community members.

## 1. Introduction

Overweight and obesity is a public health concern in Saudi Arabia. According to the Saudi Ministry of Health, nearly 60% of Saudi adults are overweight or obese [1]. Changes in the dietary habits and physical activity levels of Saudis during the COVID-19 lockdown have also been found to be associated with weight gain [2]. Indeed, the impact of obesity in Saudi Arabia was reported as costing USD 3.8 billion during 2019, representing 4.3% of the total health expenditure in Saudi Arabia that year [3].

Overweight and obesity is a prevalent health issue among adolescents in the country. In a systematic review of studies published between 2002 and 2018, the assessed prevalence of overweight and obesity among adolescents in Saudi Arabia was between 22% and 48% and showed an upward trend over the last two decades [4]. In a more recent study, a sample of more than 351,000 children and adolescents aged between two and 19 years who had visited healthcare facilities in Saudi Arabia between 2016 and 2021 showed that 20.6% could be classified as overweight or obese, with obesity found to be higher among males [5]. This increased prevalence of overweight and obesity has been associated with the increased prevalence of chronic metabolic diseases in the country [6].

Several investigations have been conducted in Saudi Arabia to understand the determinants of overweight and obesity among Saudi adolescents and children. In a study that utilized a participatory approach to measure the perspectives of students, school staff, Ministry of Education officials, and parents regarding school-related factors associated with obesity in girls aged between 13 and 15 years, it was concluded that offering affordable healthy food items (fruits and vegetables) and removing unhealthy food items (sweets and chocolate) from the school canteen, as well as more education about healthy foods, were important elements for school-based interventions aiming to reduce obesity [7].

In a study that involved a sample of 2696 students based in the south of Saudi Arabia and aged between 11 and 19 years, it was concluded that limited physical activity and exercise, either in general or during school activities, were associated with higher rates of obesity [8]. In a similar, more recent study that involved a sample of 471 male secondary school students aged between 14 and 18 years from Riyadh, Saudi Arabia, it was concluded that overweight and obese students’ activity expenditure was nearly 500 min/week lower compared to students with normal body weight [9]. Furthermore, familial influence, such as having a parental history of obesity [10] or a reduced number of siblings [11], has been found to increase the risk of having a raised BMI among Saudi adolescents.

Early recognition and management of overweight and obesity among children and adolescents are important for reducing the negative health impacts and morbidities associated with these conditions [12]. Several national and international initiatives have been implemented to tackle childhood obesity. For example, the World Health Organization’s Commission on Ending Childhood Obesity provided six main recommendations, including the promotion of healthy food intake, the promotion of physical activity, preconception and pregnancy care, early childhood diet and physical activity, and health for school-age children [13]. These efforts have been slow and inconsistent, however, as they have targeted the development of policies that require a multi-sectorial approach [14]. On a national level, the Saudi Guidelines for the Prevention and Management of Obesity were developed to provide evidence-based practices that target obesity in both adults and children [15].

The Saudi Guidelines for the Prevention and Management of Obesity provide recommendations for obesity prevention in children that include eating habit guidelines and the promotion of physical activity. The former involve specific dietary recommendations that encourage children to eat until they are full, consume regular meals including breakfast, and have a low consumption of salty and energy-dense foods. They also recommend that children consume five portions of fruits and vegetables a day, eat in sociable settings, and do not have easy access to food that is not suitable for children.

To promote physical activity in children and adolescents, the guidelines recommend reducing screentime to less than two hours a day and ensuring a minimum of one hour of active play. It is also suggested that the whole family should be involved in increasing the physical activity levels of children. If a child or adolescent is not able to maintain their body weight or prevent weight gain, referral to a specialist is advised, especially if there may be an eating disorder, poor body image, depression or anxiety, the presence of a comorbidity or underlying medical issue, or if there is a need for pharmacological or surgical intervention for weight management [15].

The Saudi Guidelines for the Prevention and Management of Obesity also recommend developing sustainable school-based interventions for proper prevention of overweight and obesity among children and adolescents. These interventions should consider an inter-sectorial approach that includes their families. Unfortunately, there is limited information regarding the involvement of schools in Saudi Arabia in the provision of school-based interventions aimed at weight management in children and adolescents. In a recent study by Al-Daghri et al. [16], which was conducted in Riyadh, Saudi Arabia, and involved a sample of 363 adolescents aged between 12 and 18 years, an intervention was developed that aimed to reduce the body weight of the adolescents by 5% or more. The intervention involved the provision of educational materials about healthy eating and the promotion of physical activity via educational sessions provided in the classroom. After 12 months, a significant reduction in BMI was found in the adherent groups compared to the non-adherent groups [16].

Although the current weight-management guidelines in Saudi Arabia recommend school-based interventions for tackling childhood obesity, the provision of such interventions is extremely limited. Furthermore, studies that assess the involvement of schools in Saudi Arabia in the promotion of healthy eating and physical activity are also limited. The contribution of schools in Saudi Arabia, via the established official educational program, towards the prevention of overweight and obesity among adolescents is currently unknown. Therefore, the current study measures the contribution of schools in the Jazan region, in the southwest of Saudi Arabia, towards the provision of education concerning the prevention of overweight and obesity among adolescents. It also identifies adolescents who have attempted to lose weight and examines their preferred weight-loss methods and their sources of weight-loss support.

## 2. Materials and Methods

### 2.1. Study Design and Settings

This investigation used a cross-sectional design to sample adolescents studying in intermediate and secondary schools in Jazan, Saudi Arabia. Data collection was initiated after securing ethical approval from the Standing Committee for Scientific Research of Jazan University (approval number REC-44/06/446, dated December 2022). Data were collected via an online platform, and an online information sheet explaining the study was shared with parents. The parents who agreed that their children could take part in the study allowed their children to complete the online questionnaire.

### 2.2. Data Collection Tool

A self-administered questionnaire collected information about the participants’ demographics, their reported body weight and height, the involvement of their schools in the provision of educational materials related to weight control, their body weight satisfaction, and the experiences of participants who had tried to lose weight. The sections of the questionnaire that measured the participants’ perceptions of their weight, their methods of weight loss, and the involvement of their schools in providing education related to weight control, were adopted from the Global School-based Student Health Survey.

The participants were asked about any education they had received concerning healthy eating, the benefits of consuming fruits and vegetables, methods for maintaining a healthy body weight, the risks of eating salty and energy-dense foods, sport in school, the benefits of physical activity, or the prevention of injury when taking part in sporting activities [17]. The participants were also asked if they were satisfied with their weight and about their intentions concerning their weight. A filtering question was used to identify participants who had tried to lose weight during the last three years, with those answering positively directed to another section of the questionnaire that examined their methods for weight loss.

### 2.3. Data Collection Process

The questionnaire was converted to an online format using Google Forms and a weblink was generated to distribute the data information sheet and the questionnaire to the parents of the participants. The parents were recruited by advertising the study in WhatsApp groups, which are a popular method of communication in school and work settings in Saudi Arabia [18].

The consenting parents were asked to give the questionnaire to their children to complete. They were also asked to share the weblink with their relatives and friends to help achieve the target sample size. Sample size estimation was conducted via the StatCal function of Epi Info. As no previous studies have used the Global School-based Student Health Survey to measure the contribution of schools towards weight management education among adolescents, it was estimated that 50% of the sample would have received relevant education. The target sample size was 480 participants, assuming a prevalence of 50% with a 5% margin of error, 95% confidence interval, and 25% refusal to participate. The expected frequency of 50% was selected for sample size estimation as it produced the maximum sample size according to the selected sample size parameters.

### 2.4. Data Analysis

Data analysis was performed using the IBM Statistical Package for the Social Sciences, version 24.0. Frequencies and proportions were used to summarize the binary and categorical data, while means, medians, standard deviations, and interquartile ranges were used to describe continuous data depending on their distribution. A standard Chi-square test investigated the relationship between BMI levels and the intention to lose weight across the sample.

BMI levels were calculated by entering the participants’ reported weight and height into the BMI Percentile Calculator for Child and Teen from the Centers for Disease Control and Prevention. The participants were then classified as underweight, normal-weight, or overweight/obese. The chi-square test looked at the relationship between the participants who had tried to lose weight in the previous three years and their BMI, body weight satisfaction, and education level. A *p*-value of <0.05 was used to indicate statistical significance.

## 3. Results

The total number of participants who completed the questionnaire was 501. The majority of the sample were female (61%) with a mean age of 16 years (SD = 1.9). More than half of the participants lived in urban areas (54%), and the majority were secondary school students (67%). When asked about their family, 86.5% of participants indicated that they were living with both of their parents, and more than half (57%) indicated that they had up to four siblings. Over half (60%) of the sample had a normal body weight, while nearly one quarter (24%) were overweight or obese. The most frequently reported medical condition was asthma (6.2%), followed by dental illnesses (5.4%). Finally, half of the sample (51%) reported that one or both of their parents had a chronic disease. See Table 1 for complete demographic information.

Table 2 shows the participants’ responses regarding the provision of education related to healthy eating and physical activity in their schools. Overall, the majority of participants (76%) reported receiving education about healthy eating. However, only 63% reported receiving education during the school year regarding how to maintain a healthy weight. Furthermore, fewer participants reported receiving education about the benefits of physical activity compared to education about healthy eating. This suggests schools in the Jazan region have low engagement in education related to the benefits of physical activity.

Table 3 displays the satisfaction of the students concerning their body weight, perception of their body weight, and their intentions concerning modifications to their body weight. More than half of the sample were satisfied with their body weight (58%), while 211 adolescents indicated that they were not satisfied (42%). When the students were asked how they would describe their body weight, the majority believed that their body weight was normal (51%). When the students were asked about their intentions concerning their body weight, nearly half of the sample indicated that they intend to modify their body weight, 22% reported that they need to make an effort to maintain their body weight, while 29% reported that they intend to do nothing about their body weight. Finally, when the adolescents were asked about their attitude about body weight, the majority indicated that body weight is important, and is important for disease prevention. However, fewer adolescents indicated that having a normal body weight would make them proud or increase their confidence.

**Figure 1:**
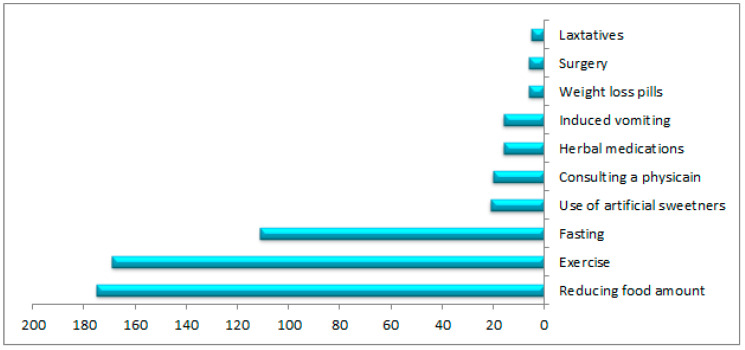
Preferences of 226 adolescents from Jazan, Saudi Arabia, regarding their weight-loss choices during the previous three years.

Slightly fewer than half of the participants (45%) reported attempting to lose weight. Figure 1 shows the frequencies of the selected methods for weight loss. The majority (77%) tried to lose weight by reducing their food consumption, exercising, or fasting. A small number of participants (9%) reported consulting a physician about losing weight, while under 10% reported using medication, artificial sweeteners, herbal medication, induced vomiting, or surgery for weight loss. Table 4 displays perceptions of weight-loss support among the participants who attempted to lose weight. The most common source of support when trying to lose weight was the participants’ families (54%), followed by community facilities (44%). The least common source of support was the participants’ schools (24%).

Table 5 shows the distribution of the participants according to their attempt to lose weight, their gender, education level, BMI category, and satisfaction with their weight. It was found that participants at secondary school, participants classified as overweight or obese, and participants who were not satisfied with their weight were more likely to have attempted to lose weight in the last three years (*p* < 0.05). Interestingly, six participants (8%) classified as underweight and 121 (41.6%) classified as normal-weight had also tried to lose weight in the last three years. This suggests low body weight satisfaction among underweight and normal-weight participants. Finally, there was no significant difference between genders in relation to weight-loss attempts.

## 4. Discussion

This study investigated the provision of education related to the prevention of overweight and obesity in adolescents and the methods used by adolescents who had attempted to lose weight. The assessment of the schools’ contributions to educating their students about weight management indicated that they were less likely to provide education about the benefits of physical activity compared to education about healthy eating habits.

Just under half of the participants reported being unsatisfied with their body weight, with a similar amount reporting that they believed they had a normal body weight. About 45% had attempted to lose weight during the previous three years, with the most common methods being reducing food consumption, fasting, and exercising. When asked about their sources of support when trying to lose weight, the most frequently reported source was their family, while the least reported source was their school. Interestingly, a number of students who were classified as underweight or normal-weight adolescents had tried to lose weight in the past three years, which suggests they had low body weight satisfaction.

There is a limited number of studies looking at the influence of schools on the eating behaviors and physical activity levels in Saudi adolescents. In a study that assessed the eating habits of 1149 elementary school students from Jeddah, Saudi Arabia, it was found that 56% consumed food and beverages from the school canteen. However, no information regarding their choice of food and beverages or their body weight was provided [19]. A similar study looked at the breakfast intake of elementary school students in Jeddah and found that 80% of the students skipped breakfast for reasons such “as not feeling hungry” and “waking up late” [20].

The current study showed that some adolescents had attempted to lose weight despite having a normal body weight or even being underweight, suggesting that they had misperceptions about their body weight. This is similar to the findings of a study by AsSaigal et al. that measured the body weight perceptions of 270 male secondary school students in the Qassim region of Saudi Arabia. They found that 35% perceived their weight incorrectly, with 18.4% of overweight and obese students perceiving their weight as normal, 53% of the underweight students also perceiving their weight as normal, and 20% of the normal-weight students perceiving themselves as overweight [21].

The attempts of adolescents who are normal-weight or underweight to lose weight can be explained by several notions related to human behavior. Adolescents, during school year and during their development, can be subjected to peer pressure and the associated body-weight shaming and stigmatization, making them more inclined to adopt harmful weight loss measures. Additionally, body weight misperception among adolescents can be influenced by the body weight norm in communities which favor individuals who are thin. Though not measured in the current study, recent evidence indicates that body image among adolescents is further affected by the impact of social media, motivating adolescents, especially girls, to weigh less and may eventually lead to the development of unhealthy eating behaviors and eating disorders [22]. This idea is supported by the findings of the current study, in which some adolescents who attempted to lose weight reported the use of induced vomiting, weight loss pills, herbal medications, and a minority of the adolescents reported consulting a physician for weight loss attempt.

Attempts to lose weight among adolescents, as well as unhealthy dieting, has been explained by several different types of factors, such as body image dissatisfaction (individual), the absence of positive adult role models (familial), and a poor school involvement (environmental) [23]. Interestingly, the adolescents in the current study showed positive attitudes toward weight loss, with the main reasons for having a normal body weight being related to health and the prevention of disease. These results are similar to the findings of a systematic review of the motivators for weight loss among adolescents, where the most common motivators were related to the desire to improve health and self-esteem [24]. Although overweight and obesity among adolescents can be managed in a clinical setting by health professionals [25], it should be noted that only a small number of the adolescents in the current study who had attempted to lose weight reported consulting a physician.

This study found that families are a more common source of support for weight loss compared to other institutions such as schools. This is similar to the results of other studies, which have shown that parental involvement can be a motivating factor for adolescents. In a study looking at 49 American adolescents who had attempted to lose weight, it was concluded that younger adolescents were more likely to be motivated to lose weight if their parents were involved compared to older adolescents [26]. The current study indicates that friends, schools, and communities are less likely to provide weight-loss support, indicating the presence of barriers to weight loss. In a review that examined weight victimization among adolescents, it was reported that this acted as a barrier to physical activity due to humiliation by others and the resulting insecurity [27].

The current study identified several different methods used by adolescents when trying to lose weight, with the most common being reducing food intake and exercising. This is similar to the findings of the American National Health and Nutrition Examination Survey, which indicated that exercising was the most frequently used method of weight loss among adolescents aged between 16 and 19 years [28]. Similarly, adolescents from Mauritius aged between 13 and 18 years were most likely to try reducing their consumption of energy-dense foods, increasing their consumption of fruits and vegetables, and exercising to lose weight [29].

The current study found that weight-loss attempts were more likely in secondary school adolescents compared to the younger, intermediary school adolescents, suggesting that age influences the likelihood of a student attempting to lose weight. Nonetheless, the study by Rancourt et al., which assessed success of weight loss initiation and maintenance among adolescents with overweight and obesity, indicated that adolescents at a younger age were more likely to initiate weight loss attempt when their parents were involved. Nonetheless, Rancourt et al. also reported that older adolescents were more likely to initiate weight loss on their own, even with less parental involvement [25]. Although there is some variability in the association between age and weight loss attempts identified in our study and the findings of the Rancourt et al. study, it must be noted that the study by Rancourt et al. was restricted to a small sample of adolescents who were overweight or obese while our study recruited a lager sample with variable levels of BMIs, including some adolescents classified as normal-weight or underweight who attempted to lose weight.

Interestingly, gender was not found to influence whether or not a student attempted to lose weight. These findings are similar to those of a large-scale European study by Dzielsa et al., which involved a sample of more than 600,000 adolescents from 26 European countries and compared weight-loss behavior in 2001/2002 to that in 2017/2018. They found that girls were more likely to engage in weight-loss behavior in 2001/2002, but that this gender difference reduced over time, with more boys attempting to lose weight during 2017/2018 [30]. In contrast, a UK-based study that compared weight-loss attempts between two periods (1997/1998 and 2015/2016) indicated that the number of these attempts increased over time and were more common among girls [31].

The current findings indicate that nearly 58% of the recruited adolescents were satisfied with their body weight. Nonetheless, among those who reported being satisfied with their body weight, 63 adolescent students (22% of the those who are satisfied) declared their wish to alter their body weight. It is possible to argue that the identified 63 adolescents may exhibit lower satisfaction levels in comparison to the majority of the satisfied adolescents who did not wish to alter their body weight. However, this was not measured in the current study since satisfaction was assessed via binary answering options (yes, no). Furthermore, satisfaction levels can be associated with different factors such as age, gender, BMI level, body composition, and appearance. This suggests the need for further research to assess satisfaction levels (such as utilizing five-point Likert scales, and to identify the factors associated with the satisfaction level and the intention to alter body weight among the adolescents).

Adolescents in Saudi Arabia spend a very significant part of their lives in schools. The education system and school environment are directly involved with the mental, emotional, and physical well-being of adolescents. It is possible to argue that any public health intervention targeting adolescents would not succeed without the optimal involvement of schools. Overweight and obesity during adolescence is rather critical due to its long-term impact and the raised risk of developing chronic non-communicable diseases during a later phase of life. Therefore, schools should invest in enhancing their communication with young people, especially during the developmental changes the adolescents are experiencing.

Enhancement of the communication of schools with young people can be implemented on several levels. Firstly, in a country that is witnessing a rise in the incidence of lifestyle-influenced chronic diseases such as obesity, diabetes, and hypertension, education about healthy eating and physical activity should be a core part of the curricular activities delivered to the students. Secondly, schools should provide tailored extracurricular activities for adolescents who are suffering from raised BMIs or are at risk of developing obesity. These curricular and extracurricular activities should be provided by teachers who themselves are adherent to a healthy lifestyle to act as role models to the adolescents, and they should be well-trained communicators with young people in a manner that addresses the emotional and psychological considerations associated with the development of adolescents. Success of the school’s communication with its adolescents via curricular and extracurricular activities should create a culture in schools that reduces stigma associated with body weight and body image and motivate the adolescents to engage in a healthy lifestyle.

The current study has several strengths and weaknesses. The main strengths are associated with the utilization of an online platform to enhance the ability to reach adolescents in different weight categories. Additionally, this study was able to identify adolescents who had attempted to lose weight without seeking help from a healthcare professional. The study’s main weaknesses are associated with the likelihood of selection bias due to its online approach and possible measurement bias in relation to the self-reported heights and weights of the participants. Nonetheless, the findings regarding the prevalence of abnormal BMIs and the presence of misperceptions about body weight among adolescents are similar to those reported in the literature locally and internationally.

Additionally, this study assessed schools’ contribution to the education of adolescents about healthy eating, physical activity, and their support of weight-loss without involving their teachers or the relevant stakeholders in the education system. Targeting the teachers and the relevant stakeholders is important to ensure objective assessment of school’s contribution to adolescents’ health. This indicates an area for further research to perform an objective assessment of the school’s contribution to a healthy lifestyle in adolescents, via assessing the opinions of the teachers and education system stakeholders concerning healthy lifestyle education among the students. Finally, this study targeted adolescents in one region in Saudi Arabia. Although the education system is unified in the country under the umbrella of the Saudi Ministry of Education, and the teaching system is similar in all regions of the country, it must be noted that the findings of the current study have limited generalizability and further research is required in other regions to assess schools’ contributions towards the education of adolescents about healthy lifestyles.

## 5. Conclusions

The findings of the current study suggest a suboptimal involvement of schools in the south of Saudi Arabia when promoting healthy eating behaviors and physical activity among students. Furthermore, some adolescents reported not being satisfied with their body weight, which partially explains why some of those classified as underweight or normal-weight had attempted to lose weight in the past three years. Among the adolescents who attempted to lose weight, the most frequently reported source of support was their family. This indicates the need to enhance schools’ contributions to supporting the adoption of healthy lifestyles. Furthermore, governmental and public agencies should establish collaboration between different sectors, such as the health, education, and municipality sectors, as well as families and communities, to tackle body weight abnormality among adolescents and enhance the adolescents’ knowledge about healthy eating, physical activity, and weight loss.

## Figures and Tables

**Table 1:** Demographic characteristics, BMI categories, and family history of chronic diseases for 501 intermediate and secondary school students from Jazan, Saudi Arabia.

Variables	Frequency [Proportion]
Gender	
Male	197 [39.3%]
Female	304 [60.7%]
Residence area	
Rural	230 [45.9%]
Urban	271 [54.1%]
Education level	
Intermediate	166 [33.1%]
Secondary	335 [66.9]
Living with the parents *	
With both parents	428 [86.5%]
With the father only	10 [2%]
With the mother only	57 [11.4%]
Number of siblings in the family	
4 or less	287 [57.3%]
More than 4	214 [42.7%]
BMI category *	
Underweight	75 [15.6%]
Normal	291 [60.4%]
Overweight or obese	116 [24%]
Diagnosis with a chronic disease	
Frequent dental illness	27 [5.4%]
Mental illness	9 [1.8%]
Obesity	21 [4.2%]
Asthma	31 [6.2%]
Sickle cell disease	13 [2.6%]
Diabetes	5 [1%]
Parental history of chronic disease	259 [51.7%]

* 19 missing cases for BMI, 6 missing cases for living with the parents’ status.

**Table 2:** Responses of 501 intermediate and secondary school students from Jazan, Saudi Arabia, regarding education in school related to healthy eating and physical activity.

Statement	Percentage Who Responded with “Yes”
During this school year, were you taught in any of your classes that healthy eating can help you be healthy and strong?	383 [76.4%]
During this school year, were you taught in any of your classes about the benefits of eating more fruits and vegetables?	350 [69.9%]
During this school year, were you taught in any of your classes how to maintain a healthy weight?	315 [62.9%]
During this school year, were you taught in any of your classes about the risks related to eating too many foods that are high in fat, sugar, or salt?	333 [66.5%]
During this school year, were you keen to take part in the sports classes provided by the school?	288 [57.5%]
During this school year, were you taught in any of your classes how to develop a physical fitness plan for yourself?	257 [51.3%]
During this school year, were you taught in any of your classes about preventing injury during physical activity?	273 [54.5%]
During this school year, were you taught in any of your classes about the benefits of physical activity?	340 [67.9%]
During this school year, were you taught in any of your classes about the opportunities for physical activities in your community?	273 [54.5%]

**Table 3:** Satisfaction with and perceptions of body weight among 501 intermediate and secondary school students from Jazan, Saudi Arabia.

Statement	Frequency [Proportion]
Are you satisfied with your body weight?	
Yes.	290 [57.9%]
No.	211 [42.1%]
How would you describe your body weight?	
My body weight is low.	72 [14.4%]
My body weight is normal.	258 [51.5%]
My body weight is high.	171 [34.1%]
Attitudes toward body weight:	
Having a normal body weight is important.	436 [87%]
Having a normal body weight is beneficial for disease prevention.	371 [74.1%]
Body weight is important for body image improvement.	373 [74.5%]
I feel proud when I have a normal body weight.	230 [45.9%]
Having a normal body weight increases confidence.	319 [63.7%]
Intentions towards their body weight:	
To do nothing.	144 [28.7%]
To increase their body weight.	60 [12%]
To reduce their body weight.	188 [37.5%]
To maintain their body weight.	109 [21.8%]

**Table 4:** Perceptions of weight-loss support among 226 intermediate and secondary school students from Jazan, Saudi Arabia.

Statement	Frequency of Agreement [Proportion]		
	Agree	Neutral	Do Not Agree
Receipt of family support.	121 [53.5]	66 [28%]	40 [17%]
Receipt of friend support.	93 [41.2%]	75 [33.2%]	58 [25.7%]
Receipt of school support.	54 [23.9%]	78 [34.5%]	94 [41.6%]
Receipt of relative and community support.	74 [32.7%]	72 [31.9%]	80 [35.4%]
Availability of community facilities supporting weight loss.	99 [43.8%]	59 [26.1%]	68 [30.1%]

**Table 5:** Associations between weight-loss attempts, gender, education level, BMI category, and weight satisfaction in 501 adolescents from Jazan, Saudi Arabia.

Variables	Attempt to Lose Weight Frequency [Proportion]		
	No	Yes	*p* Value
Gender			0.46
Male	104 [52.8%]	93 [47.2%]	
Female	171 [56.3%]	133 [43.8%]	
Education level			0.01
Intermediate	105 [63.3%]	61 [36.7%]	
Secondary	170 [50.7%]	165 [49.3%]	
BMI category *			<0.001
Underweight	69 [92%]	6 [8%]	
Normal	170 [58.4%]	121 [41.6%]	
Overweight or obese	24 [20.7%]	92 [79.3%]	
Body weight satisfaction			<0.001
Yes	215 [74.1%]	75 [25.9%]	
No	60 [28.4%]	151 [71.6%]	

* 19 missing cases for BMI.

## Data Availability

The data presented in this study are available on request from the corresponding author.
